# Carboxyl-terminal modulator protein facilitates tumor metastasis in triple-negative breast cancer

**DOI:** 10.1038/s41417-022-00559-x

**Published:** 2022-11-18

**Authors:** Cheng-Han Lin, Wen-Der Lin, Yun-Chin Huang, Yu-Chia Chen, Zhu-Jun Loh, Luo-Ping Ger, Forn-Chia Lin, Hao-Yi Li, Hui-Chuan Cheng, Kuen-Haur Lee, Michael Hsiao, Pei-Jung Lu

**Affiliations:** 1 Institute of Clinical Medicine, College of Medicine, National Cheng Kung University, Tainan, 70401 Taiwan; 2 Division of General Surgery, Department of Surgery, Kaohsiung Veterans General Hospital, Kaohsiung, 813414 Taiwan; 3 Department of Surgery, National Cheng Kung University Hospital, College of Medicine, National Cheng Kung University, Tainan, 70401 Taiwan; 4 Department of Medical Education and Research, Kaohsiung Veterans General Hospital, Kaohsiung, 813414 Taiwan; 5 Department of Radiation Oncology, National Cheng Kung University Hospital, Tainan, 70401 Taiwan; 6 Graduate Institute of Cancer Biology and Drug Discovery, College of Medical Science and Technology, Taipei Medical University, Taipei, 11031 Taiwan; 7 Genomics Research Center, Academia Sinica, Taipei, 115 Taiwan; 8 Department of Clinical Medicine Research, National Cheng Kung University Hospital, Tainan, 70401 Taiwan

**Keywords:** Breast cancer, Metastasis

## Abstract

Currently, the survival rate for breast cancer is more than 90%, but once the cancer cells metastasize to distal organs, the survival rate is dramatically reduced, to less than 30%. Triple-negative breast cancer accounts for 15-20% of all breast cancers. Triple-negative breast cancer (TNBC) is associated with poor prognostic and diagnostic outcomes due to the limiting therapeutic strategies, relative to non-TNBC breast cancers. Therefore, the development of targeted therapy for TNBC metastasis remains an urgent issue. In this study, high Carboxyl-terminal modulator protein (CTMP) is significantly associated with recurrence and disease-free survival rate in TNBC patients. Overexpression of CTMP promotes migration and invasion abilities in BT549 cells. Down-regulating of CTMP expression inhibits migration and invasion abilities in MDA-MB-231 cells. In vivo inoculation of high-CTMP cells enhances distant metastasis in mice. The metastasis incidence rate is decreased in mice injected with CTMP-downregulating MDA-MB-231 cells. Gene expression microarray analysis indicates the Akt-dependent pathway is significantly enhanced in CTMP overexpressing cells compared to the parental cells. Blocking Akt activation via Akt inhibitor treatment or co-expression of the dominant-negative form of Akt proteins successfully abolishes the CTMP mediating invasion in TNBC cells. Our findings suggest that CTMP is a potential diagnostic marker for recurrence and poor disease-free survival in TNBC patients. CTMP promotes TNBC metastasis via the Akt-activation-dependent pathway.

## Introduction

Breast cancer is the most frequently diagnosed cancer in females [1]. It is a heterogeneous disease that includes various tumor subtypes with substantial differences in their biology and clinical behavior. The five-year survival rate for breast cancer is currently >90%, but once the cancer cells metastasize to distal organs, this rate is dramatically reduced, to <30%. Triple-negative breast cancer (TNBC) accounts for 15–20% of all breast cancers; it is characterized by a typically ductal or mixed histology, with a high grade and high proliferation [2]. Relative to non-TNBC, TNBC is associated with poorer prognoses, poorer disease-free survival, and higher risk of local and distal recurrence. Once TNBC metastasizes to distal organs, the five-year survival rate decreases from 65% to 11% [3, 4]. Unfortunately, typical hormone and targeted therapies are ineffectual for TNBC because of its deficiency in the three critical receptors [5]. Furthermore, neoadjuvant chemotherapy only achieves a complete pathological response in 20–30% of patients. The development of a targeted therapy for TNBC therefore remains an urgent issue.

Protein kinase B (Akt) activation via secondary messengers generated by phosphatidyl-inositol 3-kinase (PI3K) regulates various downstream signaling molecules that modulate crucial biological responses, including cell proliferation, cell survival, protein synthesis, epithelial–mesenchymal transition (EMT)-mediated metastasis [6], and cell metabolism [7]. Akt dysregulation has been reported in a wide range of human cancers, including breast cancer [8]. Moreover, there is substantial evidence to support the hypothesis that Akt plays a vital role in tumorigenesis, with research suggesting that a combination of the constitutive activation of both Ras and Akt may induce tumor formation in mice [9]. Recently, both Keymeulen’s and Yang’s groups have reported that abnormal Akt activation can induce breast cancers to switch from luminal tumors to basal-like/triple-negative breast cancers [10] and can promote tumor metastasis via zinc finger E-box-binding homeobox 1 (ZEB1)-mediated EMT [11]. In addition, other studies suggest that Akt is more strongly activated in TNBC than in non-TNBC and is positively correlated with poorer prognoses and a higher likelihood of drug resistance [12, 13]. Therefore, studying the regulatory mechanisms of Akt activation is not only important for understanding the role of Akt dysregulation in TNBC, but is also critical to the development of therapeutic strategies against Akt overactivation in breast cancer, especially in TNBC.

Carboxyl-terminal modulator protein (CTMP) was first discovered as an Akt inhibitor in vitro and has been posited to play a role in stroke-associated neurodegeneration [14, 15]. The pathological role of CTMP in tumor progression remains controversial, however, mainly because it is based on the observation of high CTMP expression in tumor tissue relative to that in the adjacent nontumor tissue [16, 17]. Our previous studies, as well as studies from Ono’s group, have demonstrated that overexpression of CTMP can induce Akt phosphorylation, leading to increased Akt activity and facilitating apoptosis evasion, glucose metabolism, and breast cancer progression [16–18]. In our previous study, we observed CTMP upregulation in human breast cancer cell lines and specimens. CTMP expression levels were positively correlated with Akt phosphorylation and activity. Moreover, CTMP levels were inversely correlated with patient survival. The forced expression of CTMP increased basal and insulin-induced Akt phosphorylation, which facilitated cell proliferation, soft agar colony formation, and in vivo tumorigenesis. Conversely, downregulation of CTMP reduced breast cancer cell proliferation. In addition, we found that the N-terminal domain (1–64 amino acid residues) of CTMP was responsible for the Akt interaction. Combining these findings with similar results obtained by Ono’s group, we can conclude that CTMP functions as a positive regulator of Akt and plays an oncogenic role in breast cancer.

Recently, Chang and colleagues showed that CTMP triggers cell migration and cell invasion abilities in head and neck squamous cell carcinoma (HNSCC) cells and increases tumor resistance to cisplatin treatment [19]. However, whether CTMP mediates TNBC metastasis remains unclear. In this study, we provide evidence supporting the hypothesis that high CTMP levels promote TNBC metastasis via the CTMP/ Akt -dependent pathway.

## Materials and methods

### Cell culture

We used the TNBC cells MDA-MB231(ATCC Cat# HTB-26, RRID:CVCL_0062), MDA-MB157(ATCC Cat# HTB-24, RRID:CVCL_0618), HCC1599(ATCC Cat# CRL-2331, RRID:CVCL_1256), HC1806 (ATCC Cat# CRL-2335, RRID: CVCL_ 1258), HCC1937 (ATCC Cat#CRL-2336; RRID: CVCL_0290), DU44759(ATCC Cat# HTB-123, RRID:CVCL_1183), HCC70 (ATCC Cat# CRL2315, RRID: CVCL_1270), BT549(ATCC Cat# HTB-122, RRID:CVCL_1092), MDA-MB231-derived organ-tropic cells (BrM-831, LM2-4175, BoM-1833, obtaind from Dr. Joan Massagué’s laboratory) [20–22], and MDA-MB231-derived highly invasive cells (MDA-MB231 1-1, MDA-MB231 1-2, MDA-MB231 1-3, MDA-MB231 1-4, MDA-MB231 1-5) in this study. The cells were cultured in Dulbecco’s modified Eagle’s medium (DMEM; GIBCO) supplemented with 10% fetal bovine serum (FBS; HyClone) and 1% antibiotics (Antibiotic–Antimycotic solution; CORNING). The BT549 cells were cultured in RPMI medium 1640 (GIBCO) supplemented with 10% FBS, 1% antibiotics, and 0.023 IU/mL insulin. All cells were incubated in 5% CO_2_ at 37 °C.

### Clinical specimens

Human breast cancer tissue microarrays (TMA-BCs) from two clinical cohorts of patients were created from the Kaohsiung Veterans General Hospital archives. Clinical tissues were collected with informed consent and institutional review board approval (VEGHKS12-CT9-07). All tissue sections were fixed with 10% formalin, dehydrated, paraffin-embedded, and further histologically examined for the presence of tumor with hematoxylin and eosin (HE) and immunohistochemistry (IHC) staining.

### Plasmid preparation and transfection

The non-silencing shRNA vector controls, pLKO.1-shCTMP#1 (NM_053055.2-707s1c1), pLKO.1-shCTMP#2 (NM_053055.2-609s1c1), and pLKO.1 vector [23] were purchased from the National RNAi Core Facility (NRC, Academia Sinica, Taipei, Taiwan). The pLVX-dtomato-CTMP plasmid was produced by inserting the full-length cDNA for human CTMP into the vector pLVX-dtomato. pLVX-dtomato was a gift from Manuel Thery (Addgene plasmid #73332; http://n2t.net/addgene:73332; RRID:Addgene_73332). The pEGFP-C1-CTMP plasmid was produced by inserting the full-length cDNA for human CTMP into the vector pEGFP-C1. In transient transfection, 0.5 μg of plasmid DNA and 0.8 μL of PolyJet (SignaGen Laboratories, MD, USA) were each diluted with 100 μL of high-glucose serum-free DMEM. The diluted DNA and PolyJet solutions were mixed gently and incubated for 15 min at room temperature. Finally, 200 μL of the transfection complex was added into each six-well dish containing cells and 1 mL of serum-free DMEM. The cells were cultured at 37 °C in 5% CO_2_ for 8–12 h and refilled with the complete culture medium to terminate transfection. The cells were cultured in the incubator for the following experiments.

### Lentiviral vector production and transduction

The Lentiviral vectors pCMVDR8.9 (packaging plasmid), pMD.G (envelope plasmid), pLVX-dtomato-vector, pLVX-dtomato-CTMP, pLKO.1-vector, pLKO.1-shCTMP#1 (NM_053055.2-707s1c1), and pLKO.1-shCTMP#2 (NM_053055.2-609s1c1) were used to established stable CTMP-manipulated TNBC cell lines. For the lentivirus preparations, 3 × 10^6^ HEK293T cells were seeded in a 10 cm culture dish using Lipofectamine 2000, as per manufacturer’s instructions. 5 μg of the transfer plasmids, 5 μg of pCMVDR8.9, and 0.5 μg of pMD.G in Opti-MEM (GIBCO) were co-transfected into the HEK293T cells. The culture medium was renewed after 6 h and medium containing lentivirus was harvested at 24, 48, and 72 h post-transfection. The virus-containing medium was centrifuged at 1,250 rpm for 5 min at 4 °C and then passed through a 0.45 μm pore polyvinylidene fluoride filter to remove cellular debris. Lentiviral transduction was induced by adding virus-containing medium to cells and culturing overnight. Stably transduced cells were selected using puromycin, and heterogeneous pools containing fluorescent clones were sorted using fluorescence-activated cell sorting.

### Western blotting

The cells were lysed by heating 2X sample buffer (0.1 M Tris-HCI at pH6.8, 4% SDS, 20% glycerol, 2% β-mercaptoethanol, and a little bromophenol blue) and the lysates were heated for 10 min at 100°C, placed on ice for a few minutes, and then stocked at −80 °C. For immunoblotting analysis, the protein lysate per sample was loaded into 7~12% sodium dodecyl sulfate-polyacrylamide gel electrophoresis (SDS-PAGE) at 80–120 volts and then transferred the gel to polyvinylidene difluoride (PVDF) membrane (Merck Millipore, Burlington, MA, USA) at 100 volts for 90 min by using a Trans-Blot Electrophoretic Transfer Cell system. Membranes were blocked with 5% non-fat dried milk in 1X TBST (10 mM Tris, PH 7.4, 150 mM NaCl, and 0.05% Tween-20) and incubated overnight at 4 °C. Membranes were then washed three times every 10 min with 1X TBST. Membranes were incubated with the appropriate primary antibody (Supplemental Table 1), diluted by 2% bovine serum albumin (BSA) and 0.05% sodium azide, at RT for 2 h or 4 °C overnight. Membranes were then washed three times every 10 min with 1X TBST and incubated with the appropriate horseradish peroxidase (HRP)-conjugated secondary antibodies (1:10,000) at RT for 1 h and then washed four times every 10 min with 1X TBST. Detection was performed by an ECL-enhanced chemiluminescence system (PerkinElmer). The protein expression level was captured by Fuji Medical X-ray (Tokyo, Japan) and the intensities were quantified by using ImageJ (Bethesda, MD) image analysis software. The β-actin and GAPDH were regarded as the internal control for normalization.

### Migration and invasion assays

The migration and invasion assays were performed to evaluate metastatic ability in vitro, using a Boyden chamber transwell system with an 8 μm pore polyethylene terephthalate (PET) membrane (BD Biosciences, MA, USA). In the migration assay, the cells were trypsinized and suspended in serum-free medium. 1 × 10^5^ cells were resuspended with 100 μL serum-free culture medium and seeded into each transwell chamber. The transwell was transferred to a 24-well culture plate and each well was filled with 450 μL culture medium containing 10% FBS. The cells were cultured at 37 °C for 5–10 h, depending on the cell line. In the invasion assay, the membrane of the transwell was coated with 50 μL (1 mg/mL) Matrigel (BD Biosciences) at 37 °C for 2 h. The cells were cultured at 37 °C for 10–14 h, depending on the cell line. The bottom of the transwell membrane was fixed with 100% methanol for 30 min at room temperature and washed thrice with double-distilled H_2_O. Cells that had not migrated were removed with a cotton swab. The membrane was stained with 10% Giemsa reagent (Sigma, MO, USA) for 10 min and washed thrice with double-distilled H_2_O to clear the surplus dye. Finally, the cells were counted via bright-field microscopy.

### Animal studies

Four- to six-week-old female NOD/SCID mice were obtained from the National Laboratory Animal Center for xenograft studies. Animal care was provided in accordance with the Laboratory Animal Welfare Act and the Guide for the Care and Use of Laboratory Animals and was approved by the Institutional Animal Care and Use Committee of National Cheng Kung University (IACUC NO.: 106098, 107198). For establishing an orthotopic breast cancer model, 1 × 10^5^ parental MDA-MB231 cells/50 μl Hanks’ Balanced Salt Solution (HBSS) were injected into the mammary fat pads of NOD/SCID female mice. Tumor growth was detected using IVIS once a week. The animals were sacrificed at 9 weeks. The organs were then collected and subjected to IVIS detection ex vivo. For the tail-vein injection, 1 × 10^5^ cells were suspended in 30 μL of HBSS. For the intracardiac injection, 1 × 10^5^ cells were suspended in 100 μL of HBSS with ultrasound guidance. Tumor growth and metastases were monitored weekly via In Vivo Imaging System (IVIS). Organs with metastatic tumor cells were collected and evaluated using HE staining and immunochemistry.

### Hematoxylin and eosin (HE) staining

Slides containing paraffin-embedded tissue sections were deparaffinized and rehydrated. The slides were then rinsed twice with distilled water. The sections were counterstained with hematoxylin for 3 min and the slide was rinsed in tap water for 5 min. The sections were then stained with eosin for 30–60 s and the slide was rinsed in 95% ethanol for 2 min. Finally, the sections were dehydrated and mounted with mounting medium.

### Immunohistochemistry (IHC)

Slides containing the paraffin-embedded tissue sections were heated at 65 °C for 1 h, deparaffinized thrice using xylene for 10 min, and rehydrated using a graduated ethanol series (two repeats of 100%, 95%, and 75% for 5 min each), followed by a final rinse with 1× phosphate-buffered saline (PBS). Antigen retrieval was performed via immersion in 0.5 M sodium citrate buffer (pH 6.0) and boiling in a microwave for 20 min. The sample was cooled to room temperature over 30 min and then rinsed twice with 1× PBS for 5 min. Endogenous peroxidase activity was blocked via immersion in 3% H_2_O_2_/methanol for 10 min. The sample was then rinsed twice with 1× PBS for 5 min. Sections were blocked using 10% normal horse or goat serum in 1× PBS at room temperature for 30 min and then incubated with primary antibodies in antibody diluent solution at 4 °C overnight. The slides were then washed thrice with 1× PBS for 5 min and incubated with biotinylated secondary antibody (1:800). They were then washed twice with 1× PBS for 5 min, before being subjected to a standard avidin-biotin-peroxidase complex method (Vectastain Elite ABC Peroxidase Kit, Vector, CA, USA), as per the manufacturer’s instructions, at room temperature for 30 min. Immunoreaction products were visualized by applying a 3,3′-diaminobenzidine substrate (DAB, Sigma) for 5 min before they were washed twice with distilled water for 5 min. The sections were then counterstained with hematoxylin for 1 min and the slide was rinsed in tap water for 5 min. Finally, the sections were dehydrated twice in 75% ethanol, twice in 95% ethanol, and twice in 100% ethanol for 2 min at each concentration, before being immersed in xylene thrice for 5 min and mounted for evaluation.

### Gene expression microarray analysis

CTMP-overexpressing and parental MDA-MB-231 cells were collected and lysed by TRIsure^TM^. Gene expression array (SurePrint G3 Human Gene Expression 8x60K Microarray) was performed by Welgene Biotech Co., Ltd; 0.2 μg of total RNA was amplified using a Low Input QuickAmp Labeling Kit (Agilent Technologies, CA, USA) and labeled with Cy3 (CyDye, Agilent Technologies) during the in vitro transcription process. A total of 0.6 μg of Cy3-labled cRNA was fragmented to an average size of ~50–100 nucleotides by incubation with fragmentation buffer at 60 °C for 30 min. Correspondingly, fragmented labeled cRNA was then pooled and hybridized to the Agilent SurePrint Microarray (Agilent Technologies) at 65 °C for 17 h. After washing and drying by nitrogen gun blowing, the microarrays were scanned using an Agilent microarray scanner (Agilent Technologies) at 535 nm for Cy3. Raw signal data were normalized by quantile normalization for identifying differentially expressed genes.

### Statistical analysis

All observations were confirmed in at least three independent experiments. Data are expressed as means ± SD. The association between overall survival and disease-free survival was analyzed using log-rank Kaplan–Meier and Cox regression analysis. Statistical comparisons of the results were made using student *t-*tests and Mann–Whitney tests. The correlation between the level of CTMP indicated in the IHC assay and the TMN stage of the breast cancer tissue was analyzed using χ^2^ testing and Pearson’s correlation. All tests were two-sided, and *p*-values < 0.05 were considered to be statistically significant.

## Results

### High CTMP levels are correlated with recurrence and metastasis in TNBC

**Fig. 1:**
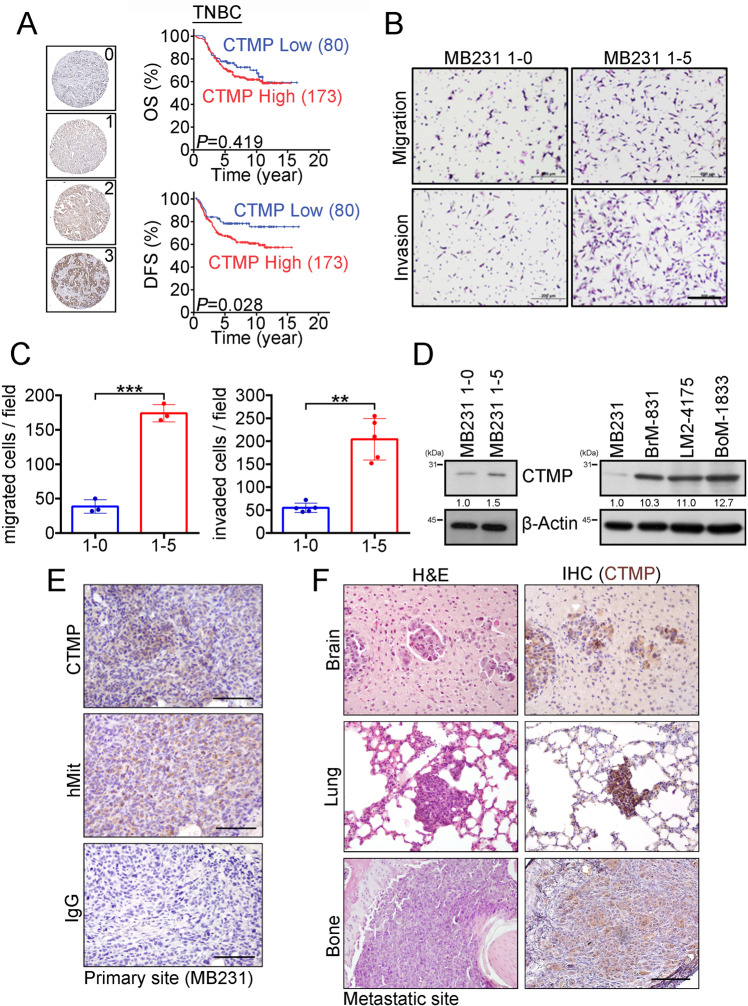
High CTMP levels are correlated with poor disease-free survival and distal metastases in TNBC. **A** CTMP expression was examined via IHC in 253 TNBC patients, and the expression of CTMP was scored at four expression levels (0–1 were combined to form the CTMP Low group; 2–3 formed the CTMP High group). Overall survival (OS) and disease-free survival (DFS) of the 253 TNBC patients were subjected to Kaplan–Meier analysis according to CTMP expression level. **B**, **C** Transwell analysis was used to evaluate the migration and invasion abilities of MB231 1-0 and 1–5 cells. **D** Western blotting demonstrated the relative expression of CTMP in highly invasive and organ-tropic MB231 cells. β-actin was used as an internal control. **E** CTMP expression was detected via IHC in xenograft tissues orthotopically injected with MB231 cells. Expression of hMit was used to differentiate the human cells in the mouse xenograft tissues. IgG was used a negative control. **F** CTMP expression was detected via IHC in brain, lung, and bone metastases in the xenograft tissues. Scale bars: 200 μm in **B**; 100 μm in E and F. **p < 0.01; ***p < 0.001.

To investigate the relationship between disease progress and CTMP expression in TNBC patients, we analyzed a TNBC tissue microarray that contained 253 TNBC specimens collected from Kaohsiung Veterans General Hospital, and conducted an IHC assay to evaluate the expression of CTMP. Each sample was scored from 0–3 according to CTMP expression levels, and the specimens were then subdivided into the CTMP Low (0 and 1) and High groups (2 and 3). We used Kaplan–Meier analysis to evaluate the associations between CTMP expression levels and overall survival (OS) and disease-free survival (DFS). Our results indicated that high CTMP levels were significantly correlated with DFS (*p* = 0.028) but not associated with OS (*p* = 0.419) in TNBC patients (Fig. 1A). We then used chi-squared analysis to investigate the relationships between various clinicopathological characteristics of the 253 patients and their CTMP expression levels. The results showed that high CTMP expression was significantly associated with recurrence (*p* = 0.014) and metastasis (*p* = 0.024) in these patients (Table 1 and Supplemental Table 2). This suggested that high CTMP expression is correlated with metastasis in TNBC patients.

**Table 1:** High CTMP is correleated with recurrence and metastasis in TNBC patients.

		CTMP expression		
		Low	High	*P*-value
**Survival status**	**Alive**	55	109	0.374
	**Death**	25	64	
**Recurrence status**	**Yes**	18	66	0.014*
	**No**	62	107	
**Metastasis status**	**Yes**	19	66	0.024*
	**No**	61	107	

* indicated *P* < 0.05

We performed five runs of an in vitro invasion selection assay to isolate highly invasive TNBC cells. In the first run, the MDA-MB-231 cells that had not invaded within 14 h were collected and referred to as MDA-MB-231 1-0 cells (MDA-MB231 1-0), and the cells that had invaded were collected and referred to as MDA-MB-231 1-1 cells (MDA-MB231 1-1). Similarly, we isolated and collected MDA-MB231 1-2, MDA-MB231 1-3, MDA-MB231 1-4, and MDA-MB231 1-5 cells, with the parental cells of each line having been selected in the previous run (Fig. S1A). We then used the transwell assay to evaluate the migration and invasion ability of MDA-MB231 1-0 and MDA-MB231 1-5 cells. We found that there were 38 ± 9 and 174 ± 12 migrated cells per field, and 55 ± 10 and 204 ± 45 invaded cells per field, among the MDA-MB231 1-0 and MDA-MB231 1-5 cells, respectively. These results indicated that the migration and invasion abilities of MDA-MB231 1-5 cells were significantly greater than those of MDA-MB231 1–0 cells (*p* = 0.0002 in the migration assay; *p* = 0.0013 in the invasion assay) (Fig. 1B, C). We then used Western blotting to evaluate the expression of CTMP in isolated cells. We found that the quantity of CTMP in MDA-MB231 1-5 cells was ~1.5 times that in MDA-MB231 1–0 cells. We also investigated the expression of CTMP in organ-tropic MDA-MB-231 cells (brain-tropic, BrM-831; lung-tropic, LM2-4175; bone-tropic, BoM-1833) [24, 25]. We found that CTMP levels were significantly higher in organ-tropic cells than in the parental MDA-MB231 cells (10.3 times in BrM-831; 11.0 times in LM2-4175; 12.7 times in BoM-1833) (Fig. 1D). To investigate the expression of CTMP in primary tumors and metastatic tissues, we orthotopically injected MDA-MB231 cells into the fat pad of NOD-SCID mice. Nine weeks after tumor cell injection, we collected the primary tumors and distal organs containing metastatic cells (Fig. S1B) and prepared tissue sections. We conducted the IHC to investigate the expression of CTMP in these xenograft tissue sections. We used immunostaining for human mitochondria to identify the human cells in the mouse xenograft tissues. We included an IHC for IgG as a negative control. The IHC of the primary tumors showed that CTMP was not homogeneously expressed, which may have been due to the heterogeneity of the tumor cells (Fig. 1E). In the distal metastatic organs, the IHC indicated that CTMP was highly expressed in metastases in brain, lung, and bone (Fig. 1F). We also investigated whether the high CTMP levels associated with metastasis in patients with TNBC possessed organ-tropic specificity. Our cohort included patients with metastases to a variety of organs and tissues, including (1) the skin and lymph nodes, (2) the supraclavicular lymph nodes, (3) the breast, (4) the lung and pleura, (5) the liver, (6) the bone, (7) the brain, (8) the chest, and (9) the adrenal glands. Chi-squared analysis indicated that high CTMP levels associated with TNBC metastasis had no organ- or tissue-tropic specificity (Table 2). Combined, these results indicate that high CTMP levels are associated with high rates of metastasis in vitro and in vivo, without organ-tropic specificity.

**Table 2:** CTMP mediated TNBC metastasis did not have organ tropism.

		CTMP expression		
		Low	High	*P*-value
**Skin and Lymph Node**	**Yes**	9	24	0.44
	**No**	10	40	
**Supraclavicular Lymph Node**	**Yes**	5	12	0.473
	**No**	14	52	
**Breast**	**Yes**	0	6	0.166
	**No**	19	58	
**Lung and Pleura**	**Yes**	10	35	0.874
	**No**	9	29	
**Liver**	**Yes**	2	14	0.051
	**No**	17	50	
**Bone**	**Yes**	6	22	0.821
	**No**	13	42	
**Brain**	**Yes**	4	9	0.462
	**No**	15	55	
**Chest**	**Yes**	0	2	0.435
	**No**	19	62	
**Adrenal Gland**	**Yes**	0	0	N/A
	**No**	19	64	

N/A indicated “Not available”

### CTMP promotes TNBC metastasis in vitro and in vivo

**Fig. 2:**
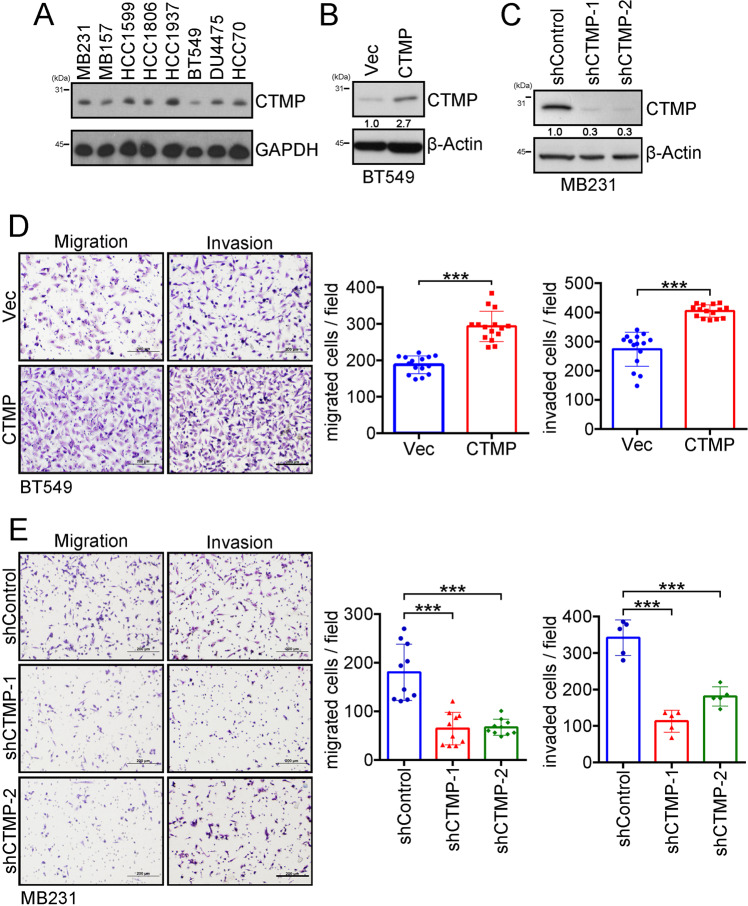
High CTMP levels promote migration and invasion in TNBC cells. **A** The expression of CTMP in various TNBC cells was examined using Western blotting. GAPDH was used as an internal control. **B** The levels of CTMP protein were investigated via Western blotting in CTMP-overexpressing BT549 cells. **C** The levels of CTMP protein were investigated via Western blotting in CTMP-downregulating MB231 cells. β-actin was used as an internal control. **D** Transwell analysis was used to evaluate the migration and invasion abilities of control (Vec) and CTMP-overexpressing BT549 cells. **E** Transwell analysis was used to evaluate the migration and invasion abilities of shControl and CTMP-downregulating (shCTMP-1, shCTMP-2) MB231 cells. Scale bar: 200 μm. ***p < 0.001.

To functionally characterize the role of CTMP in mediating the metastasis of TNBC, we manipulated CTMP expression by using plasmids containing the entire CTMP gene to upregulate it and shRNAs specifically targeting CTMP to downregulate it. We initially investigated eight TNBC cell lines and determined the mRNA level of CTMP by RT-qPCR (Supplementary Fig. 3A). We also used Western blotting to evaluate the endogenous expression of CTMP in eight TNBC cells. We found that CTMP expression was relatively low in BT549 cells and relatively high in MDA-MB231 cells. We therefore used BT549 and MDA-MB231 cells to up- or downregulate CTMP in the following experiment (Fig. 2A). We used Western blotting to evaluate CTMP expression and conducted a transwell assay to investigate metastatic ability after CTMP manipulation. We established CTMP-overexpressing BT-549 cells, which had a high CTMP mRNA expression (Supplementary Fig. 3B). We found that CTMP protein expression in BT549-CTMP was 2.7 times that in the BT549-Vec cells (Fig. 2B). We also used two independent shRNA clones (shCTMP-1 and shCTMP-2) to downregulate CTMP expression. shRNA effectively knocked down the CTMP expression at the mRNA level (Supplementary Fig. 3C) and protein level found that in MDA-MB231-shCTMP-1 and MDA-MB231-shCTMP-2 it was 0.3 times that in the MDA-MB231-shControl cells (Fig. 2C). In the migration and invasion assays, there were 187 ± 24 and 293 ± 42 migrated cells per field in the BT549-Vec and BT549-CTMP groups, respectively, and 274 ± 58 and 405 ± 21 invaded cells per field. These results indicated that CTMP overexpression significantly enhances cell migration and invasion in vitro (p < 0.001) (Fig. 2D). There were 231 ± 32, 94 ± 17, and 78 ± 17 migrated cells per field in the MDA-MB231-shControl, MDA-MB231-shCTMP-1, and MDA-MB231-shCTMp-2 groups, respectively, and 180 ± 58, 65 ± 34, and 67 ± 16 invaded cells per field in the same groups. These results indicated that CTMP downregulation significantly inhibits cell migration and invasion in vitro (p < 0.001) (Fig. 2E). These results suggest that CTMP promotes the metastasis of TNBC cells in vitro.

**Fig. 3:**
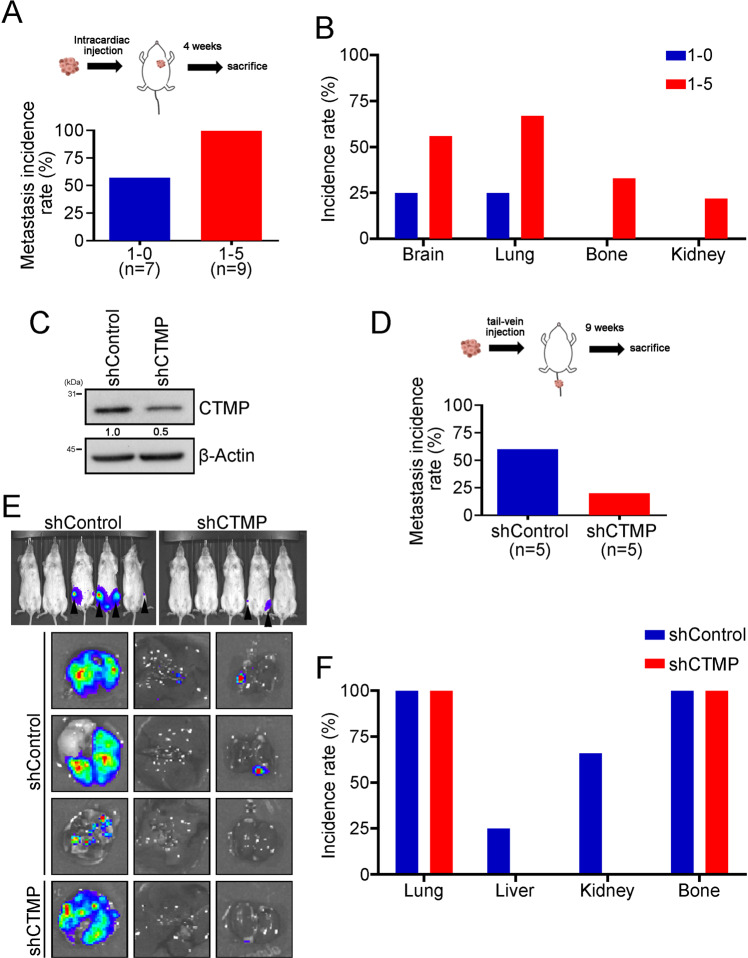
Downregulation of CTMP inhibits tumor cell colonization in vivo. **A**, **B** Four weeks after intracardiac injection of MB231 1–0 and 1–5 cells, the metastasis incidence rate and incidence rate in specific distal organs was calculated. **C** Western blotting was used to confirm stable CTMP expression in shCTMP MB231 cells. shControl and shCTMP MB231 cells were injected into NOD-SCID mice via the tail vein. An IVIS system was used to monitor tumor cell metastasis in vivo. Nine weeks after inoculation with the shControl and shCTMP MB231 cells, we assessed the (**D**) metastasis incidence rate, (**E**) distal organs with metastatic cells, and (**F**) incidence rate of metastasis to specific distal organs.

To further investigate whether high CTMP levels enhance tumor cell colonization in vivo, we inoculated NOD-SCID mice with MDA-MB231 1-0 (CTMP-low) and MDA-MB231 1-5 (CTMP-high) cells via intracardiac injection. We used an IVIS system to monitor the metastatic cells in vivo weekly. Four weeks after tumor cell injection, the mice were sacrificed and the organs with metastatic cells were identified. The metastasis incidence rate was 57.1% (4/7) and 100.0% (9/9) in the MDA-MB231 1-0 and MDA-MB231 1-5 groups, respectively (Fig. 3A). Of the mice with metastatic cells from the CTMP-high group, 55.6% (5/9), 66.7% (6/9), 33.3% (3/9), and 11.1% (1/9) experienced metastases to the brain, lung, bone, and kidney, respectively. Of the mice with metastatic cells from the CTMP-low group, only 25.0% (1/4) experienced metastases to the brain and lung (Fig. 3B). To investigate whether downregulation of CTMP inhibits tumor cell colonization in vivo, we inoculated NOD-SCID mice with MDA-MB231-shControl and MDA-MB231-shCTMP cells via tail vein injection. We used lentivirus transduction to establish MDA-MB231-shCTMP cells and used Western blotting to evaluate the CTMP expression of the established cells (Fig. 3C). We used an IVIS system to monitor the metastatic cells in vivo weekly. Nine weeks after tumor cell injection, we calculated the metastasis incidence rate, which was 60.0% (3/5) and 20.0% (1/5) in the MDA-MB231-shControl and the MDA-MB231-shCTMP groups, respectively (Fig. 3D). Of the mice with MDA-MB231-shControl metastatic cells, 100.0% (3/3), 33.3% (1/3), 66.7% (2/3), and 100.0% (3/3) experienced metastases to the lung, liver, kidney, and bone, respectively. Of the mice with MDA-MB231-shCTMP metastatic cells, 100.0% (1/1) experienced metastases to the lung and bone (Fig. 3E, F). These results indicated that the downregulation of CTMP decreased the incidence rate of metastases and distant colonization in vivo. Combined, our data suggest that CTMP promotes TNBC metastasis both in vitro and in vivo.

### CTMP enhances TNBC cell invasion via an Akt-dependent pathway

**Fig. 4:**
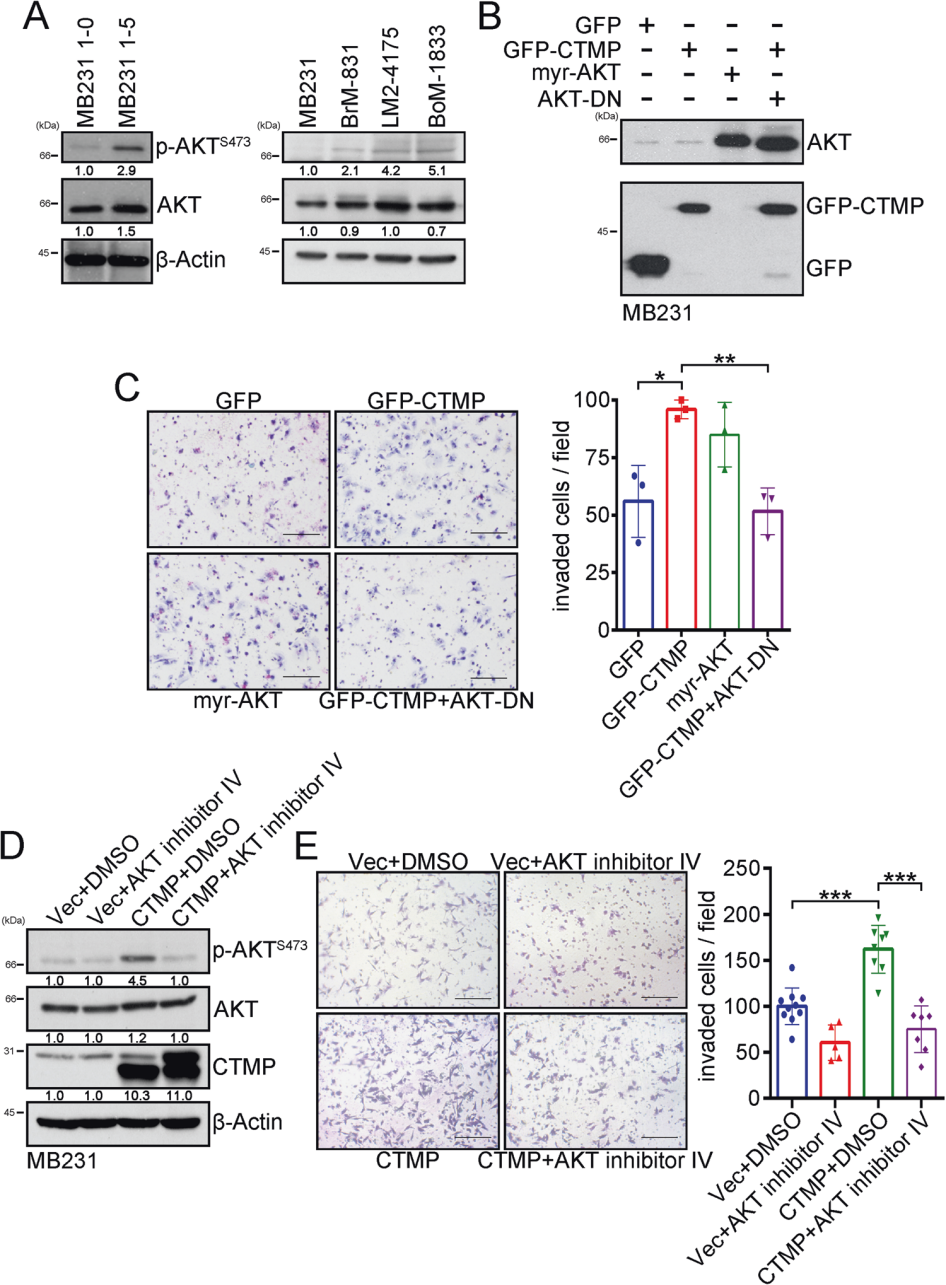
Overexpression of CTMP enhances TNBC cell invasion via a CTMP/Akt activation-dependent pathway. **A** Protein levels of p-Akt^S473^ and Akt were investigated via Western blotting in somewhat invasive (MB231 1–0), highly invasive (MB231 1–5), and organ-tropic MB231 cells. **B** The expression of Akt and GFP was evaluated via Western blotting in MB231 cells transiently transfected with (i) GFP; (ii) GFP-CTMP; (iii) myr-Akt; or (iv) GFP-CTMP and Akt-DN plasmids. **C** Transwell analysis was used to evaluate the invasion abilities of MB231 cells transiently transfected with (i) GFP; (ii) GFP-CTMP; (iii) myr-Akt; or (iv) GFP-CTMP and Akt-DN plasmids. **D** The protein levels of p-Akt^S473^, Akt, and CTMP were investigated via Western blotting in MB231 cells transfected with control and CTMP plasmids treated with Akt inhibitor. β-actin was used as an internal control. **E** Transwell analysis was used to evaluate the invasion abilities of MB231 cells treated with (i) Vec+DMSO; (ii) Vec+Akt inhibitor IV; (iii) CTMP; or (iv) CTMP + Akt inhibitor IV. Scale bar: 200 μm. *p < 0.05; **p < 0.01; ***p < 0.001.

To investigate the underlying mechanisms by which CTMP mediates TNBC metastasis, we performed RNA sequencing to identify potential signaling pathways enhanced by the overexpression of CTMP in MDA-MB231 cells relative to the parental cells. We grouped the potential signaling pathways according to the following databases: (1) KEGG pathway; (2) molecular functions; (3) biological processes; (4) cellular components (Fig. S2). In addition, in our previous study, we found that the 1–64 N-terminal amino acid residues of CTMP interact with the regulatory domain of Akt to promote breast cancer tumorigenesis [14]. Our results also showed that the PI3K/Akt pathway is significantly activated in MDA-MB231 cells overexpressing CTMP, especially relative to the parental cells (Fig. S2). We then hypothesized that high CTMP levels may mediate TNBC metastasis via the Akt-dependent signaling pathway. Western blotting indicated that MDA-MB231 1-5 cells contained 2.9 times the quantity of p-Akt ^S473^ found in MDA-MB231 1-0 cells. Expression of p-Akt ^S473^ in BrM-831, LM2-4175, and BoM-1833 cells was also 2.1 times, 4.2 times, and 5.1 times that in parental MDA-MB231 cells, respectively (Fig. 4A). These results indicated that activation of the Akt pathway is associated with highly metastatic cells. We then used two-way demonstration methods to evaluate the effects of active and inactive Akt signaling on cell invasion. We transiently transfected plasmids with green fluorescent protein (GFP) (control), GFP-CTMP, myr-Akt (constitutively active form of Akt), and Akt-DN (dominant negative form of Akt) into MDA-MB231 and CTMP-overexpressing MDA-MB231 cells. We conducted a transwell assay to evaluate the invasion ability of the transfected cells. We found that the number of invaded cells per field was 56 ± 15, 96 ± 4, 85 ± 14, and 51 ± 10 in the control, GFP-CTMP, myr-Akt, and GFP-CTMP + Akt-DN groups, respectively. Overexpression of CTMP and myr-Akt significantly increased cell invasion ability relative to the control group (*p* = 0.04 and *p* = 0.08, respectively), and cells transfected with the GFP-CTMP plasmid had a significantly reduced cell invasion ability when co-transfected with the Akt-DN plasmid (*p* = 0.009) (Fig. 4B, C). We then used the Akt inhibitor, Akt inhibitor IV, to block Akt signaling. Western blotting indicated that the expression of p-Akt ^S473^ in CTMP-overexpressing cells was 4.5 times that in the vector control cells. After inhibitor treatment, the expression of p- Akt ^S473^ decreased to the basal levels found in control cells (Fig. 4D). In addition, we found that the number of invaded cells per field was 100 ± 20, 42 ± 13, 162 ± 26, and 75 ± 25 cells in the vector control, vector control/Akt inhibitor IV, CTMP, and CTMP/Akt inhibitor IV groups, respectively. The number of invaded cells was significantly higher in the CTMP-overexpressing group (*p* < 0.001), but significantly lower in Akt inhibitor IV-treated CTMP-overexpressing cells, than in the untreated control (*p* < 0.001) (Fig. 4E). These results show that CTMP enhances TNBC invasion via an Akt-dependent pathway.

## Discussion

Metastasis is a dynamic process involving the movement of cancer cells from their primary site and their colonization of distal organs. Targeting cancer metastasis is a critical issue for cancer therapy because it is correlated with a high mortality rate in various types of cancer, including TNBC [26]. Therefore, identifying potential diagnostic molecules that are correlated with TNBC recurrence and metastasis and revealing the underlying mechanisms by which these molecules mediate TNBC metastasis may help scientists and physicians to develop novel therapeutic strategies for TNBC patients. In previous study, we demonstrated that CTMP is an oncogenic driver in breast cancer and positively regulates Akt phosphorylation [16]. In this study, we further demonstrated that CTMP promotes TNBC metastasis via an Akt-activation-dependent pathway. High CTMP levels were correlated with TNBC recurrence and disease-free survival. The expression of CTMP was increased in highly invasive and organ-tropic TNBC cells both in vitro and in vivo (Fig. 1 and Tables 1, 2). Two-way demonstration indicated that CTMP promotes tumor cell migration and invasion in TNBC cells (Fig. 2). Downregulating CTMP expression in TNBC cells successfully inhibited the metastasis incidence rate in vivo (Fig. 3). Akt pathway activation was significantly increased in highly invasive and organ-tropic TNBC cells. Blocking Akt activation successfully inhibited CTMP from mediating TNBC cell invasion (Fig. 4). Based on these results, targeting the CTMP/Akt pathway may be a potential strategy for preventing TNBC metastasis.

Chang and colleagues demonstrated that CTMP functions as a positive regulator of Akt in HNSCC. In HNSCC, higher CTMP levels are correlated with poorer overall survival, disease-free survival, and lymph node metastasis. CTMP promotes HNSCC cell invasion by regulating EMT in a Snail-dependent manner [19]. We obtained similar results in our present study. We conducted qRT-PCR to identify the EMT-related genes that were expressed after CTMP overexpression in TNBC cells. The expression of N-CADHERIN, VIMENTIN, and SNAIL in CTMP-overexpressing TNBC cells was 2.3 times, 2.5 times, and 9.4 times that in the control group, respectively (data not shown). Akt signaling activation increases in HNSCC. In this study, we have further demonstrated that Akt signaling activation is critical for the functioning of CTMP. The dominant-negative form of Akt and the Akt inhibitor were able to abolish TNBC invasion mediated by CTMP (Fig. 4). These results further supported our hypothesis that CTMP may be a risk factor for the development of distal metastases and disease recurrence in various kinds of cancer.

TNBC is more aggressive than other breast cancer subtypes and is associated with a poorer prognosis because there are fewer targeted therapies for it [27]. So far, several target reagents have been offered as novel therapeutic options for the treatment of TNBC; however, the results from these clinical trials are limited [28]. Our previous study demonstrated that CTMP promoted trastuzumab resistance via the activation of Akt signaling in HER2-enriched breast cancer cells [29]. In an associated experiment, we also demonstrated that CTMP overexpression enhances cisplatin, docetaxel, and epirubicin resistance in TNBC cells (in prep.). The identification of small molecules specifically targeting CTMP or disrupting the interaction of CTMP with Akt may therefore represent an opportunity for the development of a novel treatment for TNBC.

The purpose of this study is to characterize the functional role of CTMP that specifically associated with poor disease-free survival in TNBC and determine the underlying signaling pathway by which CTMP contributes to metastasis. Overall, we provided solid evidence that CTMP promotes TNBC metastasis via an Akt-activation-dependent pathway. High CTMP were significantly associated with TNBC recurrence and disease-free survival. Two-way demonstration indicated that CTMP promotes metastasis in vitro and in vivo. Blocking Akt activation successfully abolished the CTMP mediating invasion in TNBC cells. According to the above results, targeting CTMP/Akt pathway may be a potential strategy for preventing TNBC metastasis.

## Supplementary information

Supplemental information

## Data Availability

All data generated or analyzed during this study are included in the published article and its supplementary information files.
