# Supplementary material for: Carboxyl-terminal modulator protein facilitates tumor metastasis in triple-negative breast cancer
Source: Cancer Gene Ther. 2022 Nov 18;30(3):404–13. doi: 10.1038/s41417-022-00559-x (PMC10014580; doi:10.1038/s41417-022-00559-x)

**Supplemental information**

**Supplemental Table 1: List of antibodies used in the study**

**Supplemental Table 2: The clinical information of 253 TNBC patients**

**Supplemental Figure legends**

**Figure S1.** A. The *in vitro* invasion selection system. The diagram shows the selection system. The un-invasive (MB231 1-0) and highly invasive (MB231 1-5) cells were selected from the parental MB231 cells. B. MDA-MB231 cell line was fat-pad injected into NOD-SCID mice. After 9 weeks, the organs were collected and detected through IVIS system *ex vivo*.


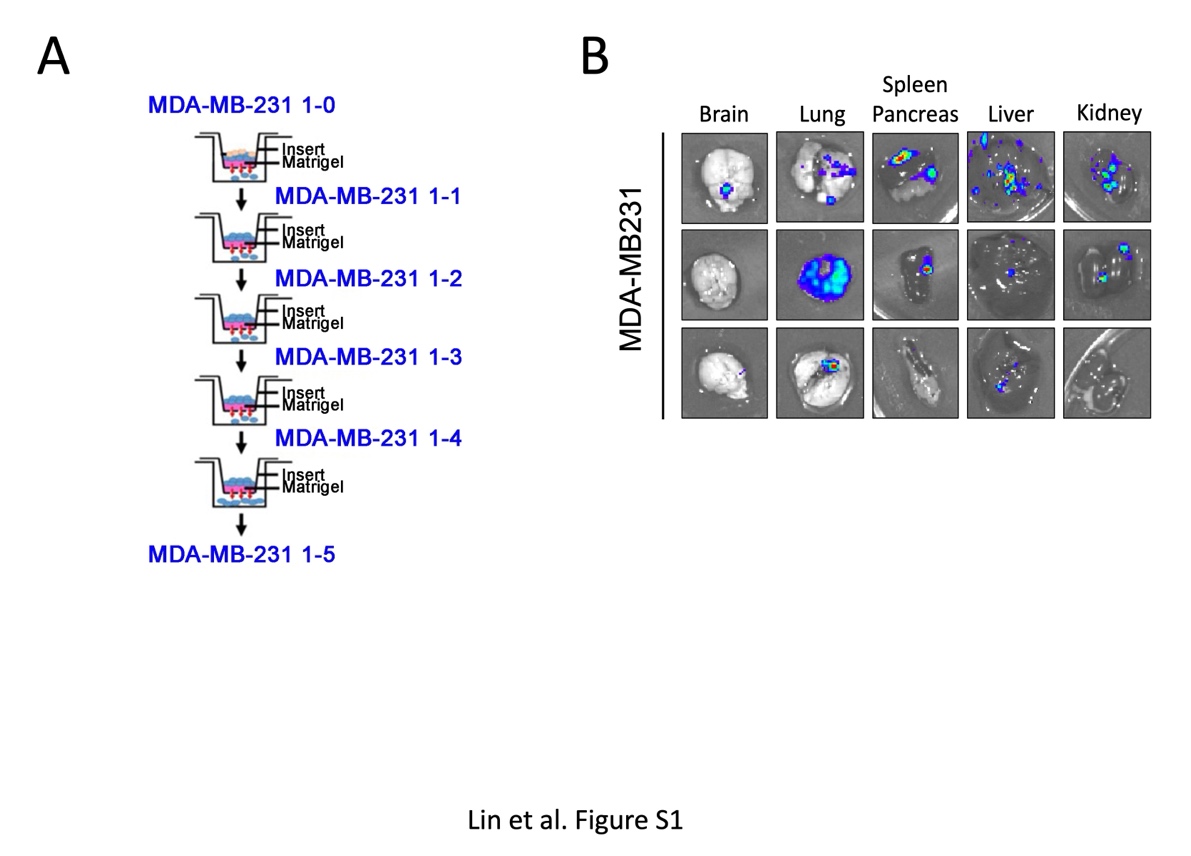


**Figure S2. The RNA sequencing analysis to reveal the differential expression pathways in MB231-CTMP compared with the parental MB231 cells.** The analysis of KEGG pathway, cellular component, biological process, and molecular function significantly enhanced in CTMP overexpression MB231 cells compared to the parental cells.


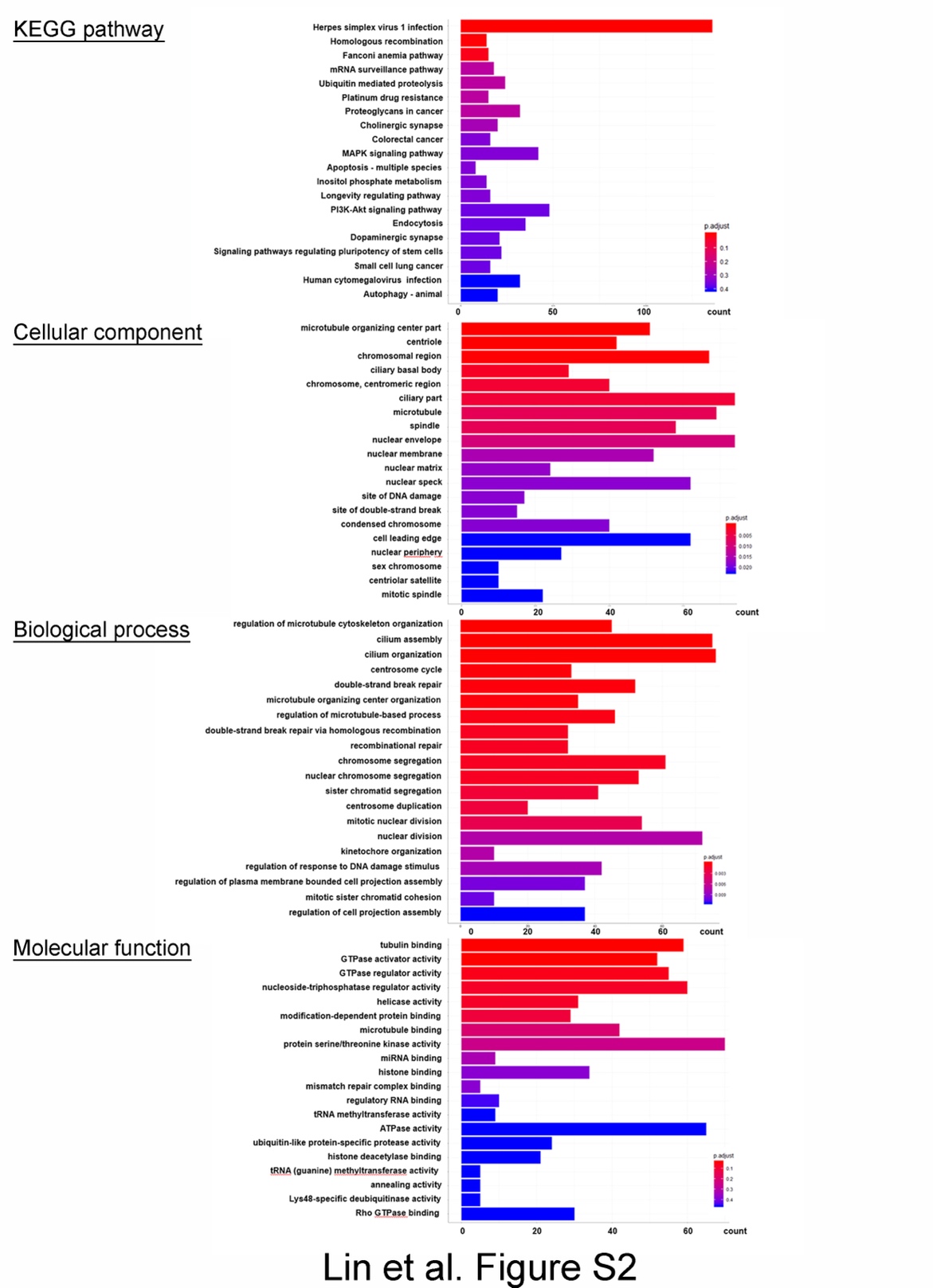


**Supplementary Figure 3. RT-qPCR analysis of various TNBC cells, CTMP overexpression BT549 cells, and CTMP knockdown MDA-MB231 cells.**

A. RT-qPCR analysis of CTMP mRNA in the indicated breast tumor cell lines and the RT-qPCR results were normalized to MDA-MB231. Error bars, SEM. n=3. B. RT-qPCR analysis of CTMP expression in control and CTMP overexpression cells (BT549), or (C) control and sh-CTMP cells (MDA231). Error bars, SEM. n=3. ****p＜0.0001 by Student’s t test.


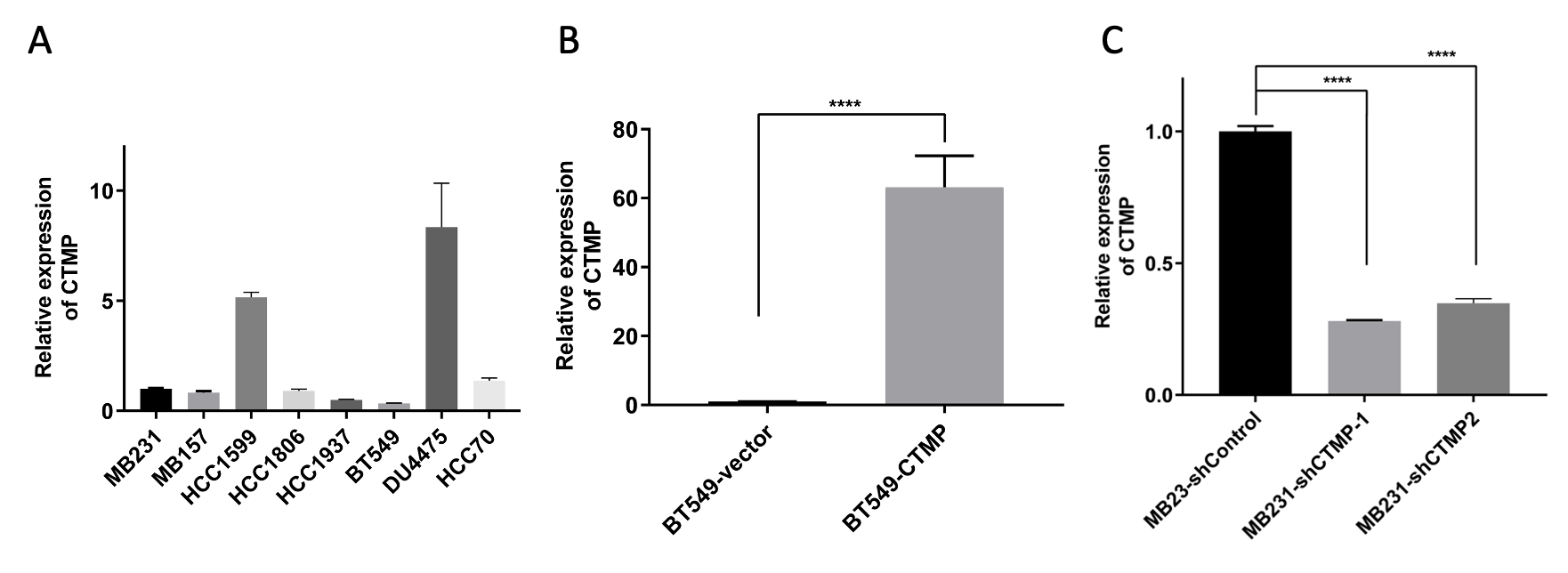

Supplement: Supplementary file 1 — Supplemental information [file 41417_2022_559_MOESM1_ESM.docx]
